# Translation of the Children’s Visual Function Quality of Life instrument from English to Sepedi

**DOI:** 10.4102/hsag.v30i0.3035

**Published:** 2025-09-26

**Authors:** Tshubelela S.S. Magakwe, Rekha Hansraj, Zamadonda Xulu-Kasaba

**Affiliations:** 1 Department of Optometry, Faculty of Health Sciences, University of KwaZulu-Natal, Durban, South Africa

**Keywords:** children, instrument translation, quality of life, reliability, validation, visual function quality of life

## Abstract

**Background:**

The Children’s Visual Function Quality of Life (CVF-QoL) instrument assesses how visual impairment affects the quality of life related to visual function among rural school-going children in South Africa. Currently, there are no validated vision tools for Sepedi-speaking children.

**Aim:**

This study aims to translate the CVF-QoL instrument into Sepedi.

**Setting:**

Three rural schools in Sekhukhune district, Limpopo, South Africa.

**Methods:**

This study utilised a forward-backward-forward translation method. Three independent translators were involved in translating the English versions of the CVF-QoL instrument into Sepedi. The translated Sepedi version was face-validated by learners aged 6–17 years. The final Sepedi versions were administered to children in two groups: version 1.1 for ages 6–9 years and version 2.1 for ages 10–17 years. The same learners completed the questionnaire again 10 days after the initial administration.

**Results:**

Face validation indicated that both versions are clear and relevant. Version 1.1, administered to 41 learners aged 10–17, achieved a Cronbach’s alpha of 0.930. Version 2.1 was given to 39 learners aged 6–9, scoring 0.762. Test–retest reliability showed scores of 0.927 for version 1.1 and 0.712 for version 2.1 after 10 days. Overall, the Cronbach scores for the Sepedi and English versions of 1.1 were 0.930 and 0.946; for version 2.1, the scores were 0.762 and 0.777.

**Conclusion:**

Sepedi version of the CVF-QoL instrument, focused on rural South African children, is novel, valid and reliable.

**Contribution:**

The translated tools can be used to evaluate the visual function of Sepedi-speaking children.

## Introduction

Visual impairment (VI) can have a considerable negative impact on the educational achievement, social interactions and emotional well-being of children, often leading to additional associated economic costs (Atowa, Hansraj & Wajuihian 2019; Tadić et al. 2016). Early detection and intervention in children with VI are thus crucial for maximising visual potential and overall development (Aboobaker & Courtright 2016; Flanagan, Jackson & Hill 2003). Additionally, VI significantly affects the quality of life of affected children (Elsman et al. 2021).

Recently, there has been increased interest in developing and utilising self-reported outcomes related to quality of life and patient satisfaction, specifically for vision-related issues (Khadka et al. 2010). These outcomes are referred to as vision-related quality of life (VRQoL) tools. Vision-related quality of life refers to how much an individual’s vision affects their ability to perform daily activities and influences their socioeconomic, emotional and social well-being (Ekemiri et al. 2023). In many cases, an eye examination on a child relies on the eye clinical findings of the eye care professional often supplemented by information from parents, guardians or teachers (Angeles-Han et al. 2011). Furthermore, it remains questionable as to whether the clinical tests performed can accurately capture the child’s lived experience with VI (Angeles-Han et al. 2011). Therefore, additional input from an assessment of quality of life is crucial as many children may struggle to clearly articulate what issues they are experiencing because of their VI (Gothwal, Lovie-Kitchin & Nutheti 2003).

Several studies have examined VRQoL tools for children with VI. For example, the Children’s Visual Function Questionnaire (CVFQ) was utilised to measure VRQoL in children with bilateral congenital cataracts, revealing lower scores across all subscales compared to the control group (Lopes et al. 2009). Other instruments, such as the Cardiff Visual Ability Questionnaire for Children (CVAQC) (Khadka et al. 2010), the Impact of Vision Impairment for Children (IVI-C) (Cochrane et al. 2011) and the Pediatric Quality of Life Inventory (PedsQL) (Lamoureux et al. 2010), have also been used to assess functional visual ability and VRQoL in children with glaucoma and cataracts (Dahlmann-Noor et al. 2018).

Researchers have also developed new VRQoL instruments, including the EYE-Q for juvenile idiopathic arthritis-associated uveitis (Angeles-Han et al. 2011) and the VQoL_CYP for visually impaired children (Tadić et al. 2016). A child-centred approach has led to the creation of age-appropriate extensions of the VQoL_CYP for children aged 8 to 17 years (Rahi et al. 2011; Tadić et al. 2020). The World Health Organization’s Quality of Life (WHOQOL) group has suggested that each country should create its own tool to measure the impact of diseases and treatments (The WHOQOL Group 1995). This tool should be culturally sensitive, age-appropriate and available in the country’s official languages (The WHOQOL Group 1995). A scoping review conducted in 2024 of published literature on VRQoL tools concluded that there is no quality of life instrument specifically designed to assess visual function for African school-going children (Magakwe, Hansraj & Xulu-Kasaba 2024). For these rural school-going children instead, there is reliance on tools developed for children in developed countries, which may include items that are irrelevant to rural school-aged children. Important daily activities that these children engage in, such as tending to cattle, fetching water from a river and walking on gravel roads, are frequently overlooked. To address this gap, the Children’s Visual Function Quality of Life (CVF-QoL) instrument was developed (Magakwe, Hansraj & Xulu-kasaba 2025).

The CVF-QoL instrument was developed to measure the impact of VI on quality of life for rural school-going children within a South African context (Magakwe et al. 2025). The instrument has two versions in English: version 1.1 for children aged 10 to 17 years and version 2.1 for younger children aged 6 to 9 years. It is recommended that the quality of life instrument be translated into the first language of those individuals for whom it was designed to enhance its understanding and relevance (Guillemin, Bombardier & Beaton 1993; Marsh & Truter 2021). Therefore, this study aimed to translate the validated English versions of the CVF-QoL Instrument into Sepedi and to validate the translated instrument. Sepedi is one of the official languages of South Africa and is spoken by 12.4% of the country’s population (UNFPA South Africa 2018). It was selected because the CVF-QoL instrument was developed and validated by children from the Sekhukhune area, where Sepedi is the dominant language, spoken by 83% of the local community (Alkalah 2016).

This translation is crucial for assessing the impact of VI on children’s daily lives across different cultures and languages. Such translations facilitate cross-cultural comparisons and provide valuable insights into patient-reported outcomes (Pérez-Mañá et al. 2019). At present, there are no validated quality-of-life instruments specifically designed within a rural South African context. This absence of appropriate tools poses significant challenges, as language barriers can hinder individuals’ ability to accurately report their experiences and difficulties related to VI (Guillemin et al. 1993; Marsh & Truter 2021). Consequently, this limitation can adversely affect the quality of service delivery for children living with VI, making it difficult for healthcare providers to understand their needs and deliver the most effective interventions. The translation of this tool can also serve as a guide towards the development of similar tools in all the official languages in South Africa.

## Methodology

This translation and validation study was conducted among rural, school-going children with VIs who resided in the Sekhukhune district in Limpopo province, South Africa.

### Instrument description

The CVF-QoL instrument includes two versions. The English version 1.1 contains 89 items and covers eight domains, while the English version 2.1 consists of 63 items and also addresses eight domains. The English version 1.1 was designed for children aged 10–17, while version 2.1 was intended for younger children aged 6–9. Detailed descriptions and information on both English versions, including their rating scales and scoring information, have been published and can be accessed via this link https://doi.org/10.3390/diagnostics15030331.

### Translation process

The two versions of the CVF-QoL instrument were translated from English to Sepedi using the forward-backward-forward method as outlined by the International Quality of Life Assessment group of the World Health Organization (WHO) (Gandek & Ware 1998). The following steps were taken in translating and validating the Sepedi versions of the CVF-QoL instrument:

#### Forward translation

The English versions of the CVF-QoL instruments were sent to two independent translators. One translator was a lecturer at the University of Limpopo who taught the Sepedi module, while the other was from Afrolingo Translation Services, a South African company that translates over 100 national and international languages. Both translators were native Sepedi speakers and were tasked with translating the English versions 1.1 and 2.1 into Sepedi. After completing the translations, the reports from both translators were merged by a principal investigator (PI), whose native language is also Sepedi, resulting in one combined Sepedi version 1.1 and one combined Sepedi version 2.1.

#### Backward translation

The combined Sepedi version 1.1 and the combined Sepedi version 2.1 were sent to a third translator, a qualified language practitioner for both English and Sepedi languages, who translated them back into English. The back-translated versions were sent back to the PI to compare them to the original English versions.

#### Expert review

The PI and the translators met via a Microsoft Teams meeting to address any discrepancies observed between the original English versions, forward and backward translated versions. This was done to ensure that the Sepedi versions aligned with the English content. The meeting resulted in a final forward translation with no discrepancies between the English and Sepedi versions of the CVF-QoL instruments.

#### Pilot testing

The final versions of the Sepedi instrument underwent face validation by presenting them to 30 learners. All the selected learners were native Sepedi speakers and were learning Sepedi as their first language in school. The Sepedi version 1.1, aimed at school children aged 10 to 17 years, was validated by 15 learners within that age group. Meanwhile, the Sepedi version 2.1 was validated by 15 learners aged 6 to 9 years. All learners came from Leduma Primary School and Moreko High School, which were purposively sampled because of their easy access by the PI and because the children were learning Sepedi as a home language. Their role was to assess the clarity, formatting and relevance of the items. Items were retained if more than 70% of the learners indicated that they understood the task and found it important. Modifications were made based on the feedback from the learners, resulting in the final Sepedi versions of the CVF-QoL instrument. Online Appendix 1 shows an example of the rubric used for learners when evaluating the items and the overall instrument.

### Validation process

To evaluate the reliability and validity of the final CVF-QoL Sepedi versions, the two instruments were administered to a different group of learners from those who participated in pilot testing. While the initial expected sample size was 32 participants per Sepedi version, 40 participants were recruited for each version to account for potential dropouts. Thirty-nine learners aged 6 to 9 years completed Sepedi version 2.1, while another group of 41 learners aged 10 to 17 years completed Sepedi version 1.1. The learners were randomly selected from four schools in the Sekhukhune district, which were chosen purposefully for easy access to the PI, and the children in those schools were learning Sepedi as their home language. The schools included Leduma Primary School, Jane Furse Comprehensive School, Moreko High School and Bosele School for the Blind.

The learners underwent a series of eye examinations, which included assessments of visual acuity, visual fields, cycloplegic auto-refraction and fundus examination. Participants were included if they had been diagnosed with refractive error, VI or blindness. Moreover, included learners were those who were able to read fluently. Learners who were normally sighted or aged below 6 or above 17 were excluded from the study. On completion, each learner was given an additional questionnaire to complete at home 10 days later. This approach was also utilised in other studies that developed similar instruments for children in different contexts (Andersen 2013; Angeles-Han et al. 2011; Robertson et al. 2020). This was done to determine the stability of the Sepedi version of the CVF-QoL instrument.

### Data analysis

For data analysis, Cronbach’s alpha with a 95% confidence interval was calculated for both final Sepedi versions under the guidance of a statistician. An alpha score below 0.6 indicates fair internal consistency, scores between 0.6 and 0.8 indicate good internal consistency and scores above 0.8 indicate excellent internal consistency (Sauza et al. 2017).

### Ethical considerations

The study adhered to the ethical principles outlined in the Declaration of Helsinki for research involving human participants and received approval from the Biomedical Research and Ethics Committee (BREC) at the University of KwaZulu-Natal (BREC/00003939/2022). Consent and assent forms were completed by the parents and children, respectively. The Ethics Committee of the Limpopo Department of Education also approved this study (Ref: 2/2/2).

## Results

Each translator had the opportunity to translate version 1.1 and version 2.1 of the CVF-QoL instrument. The reports from two translators for version 1.1 were combined to create a single Sepedi version 1.1 instrument, while the reports for version 2.2 were similarly merged into Sepedi version 2.2 by the PI. The Sepedi version 1.1 contained 89 items, whereas the Sepedi version 2.2 had 63 items.

Each Sepedi version underwent face validation with 15 learners aged between 6 and 17 years, whose demographics are detailed in Table 1. The learners rated all content and items of the CVF-QoL instrument as clear, comprehensive and relevant. They also provided input on word choices, indicating a preference for ‘raloka’ over ‘bapala’, and noted the omission of a game called ‘Wulu’, which they suggested should be included.

**TABLE 1:** Demographics of the learners who participated in the face validation for both versions of the Children’s Visual Function Quality of Life instrument.

Variables	Version 1.1 participants		Version 2.1 participants	
	Number	%	Number	%
**Age in years**				
6–7	0	-	6	40
8–9	0	-	9	60
10–12	4	27	0	-
13–15	6	40	0	-
16+	5	33	0	-
**Gender**				
Males	5	33	7	47
Females	10	67	8	53
**Grades**				
1–3	0	-	9	60
4–6	3	20	6	40
7–9	7	47	0	-
10–12	5	33	0	-

As a result of the face validation, both Sepedi versions of the CVF-QoL were modified, leading to the final versions presented in Online Appendix 2 and Online Appendix 3. These final versions were then subjected to further validation.

Table 2 outlines the rating scales of the two Sepedi versions, which are Online Appendices 1 and 2, and shows how the values for each rating scale were derived. For example, the question ‘Indicate how much difficulty you have with the following activities due to vision problems’, translated to ‘Bontšha gore go bothata gakakaang go wena ka ditiragalo tše di latelago ka lebaka la mathata a go bona ga gago’, the responses can be rated as follows:

- 3 (Go thata kudu) with a score of 0
- 2 (Go thatanyana) with a score of 50
- 1 (Ga go thata) with a score of 100
- 0 (Ga ke amege mo), scored as ‘#’ to indicate a missing response. This score should not be included when calculating the average score.

**TABLE 2:** Rating scales and values for the Children’s Visual Function Quality of Life instrument.

Options	Change the original response category	To record the value of:
**Bontšha gore go bothata gakakaang go wena ka ditiragalo tše di latelago ka lebaka la mathata a go bona ga gago:**		
Go thata kudu	3	0
Go thatanyana	2	50
Ga go thata	1	100
Ga ke amege mo	0	# (missing)
**Hle bontšha ge e ba dilo tše di a go diragalela:**		
Ee	3	0
Ka dinako tše dingwe	2	50
Aowa	1	100
Ga ke amege mo	0	#(missing)
**Bontšha gore se se direga makga a makae:**		
Ka dinako ka moka	3	0
Ka dinako tše dingwe	2	50
Ga se ke se direge	1	100
Ga ke amege mo	0	#(missing)
**Naa go bothata goba bonolo bjang go wena go dira dilo tše di latelago ka lebaka la bothata bja mahlo a gago:**		
Go bothata kudu go nna	3	0
Ga go bonolo go nna	2	50
Go bonolo kudu go nna	1	100
Ga ke dire seo	0	# (missing)
**Hle bontšha ge e ba dilo tše di a go diragalela:**		
Ee	2	0
Aowa	1	100
Ga ke amege mo	0	#(missing)
**Laetša gore se se direga makga a makae:**		
Dinako ka moka	3	0
Dinako tše dingwe	2	50
Ga e direge	1	100
Ga ke amege mo	0	#(missing)

Scores indicate the percentage achieved of the total possible score; for example, a score of 50 indicates 50% of the highest possible score.

Forty-one learners aged 10 to 17 years completed the Sepedi version 1.1 of the CVF-QoL instrument. The majority of them were female (61%), and their visual status is outlined in Table 3. In total, 39 learners aged 6 to 9 years completed the Sepedi version 2.1 of the CVF-QoL questionnaire, with a higher number of females. Their demographics are detailed in Table 2.

**TABLE 3:** Demographic information of participants who completed both Sepedi Versions of the Children’s Visual Function Quality of Life instrument.

Variables	Version 1.1 participants		Version 2.1 participants	
	Number	%	Number	%
**Ages in years**				
6–7	0	-	15	38.5
8–9	0	-	24	61.5
10–12	7	12	0	-
13–15	22	54	0	-
16+	12	29	0	-
**Gender**				
Females	25	61	24	61.5
Males	16	39	15	38.5
**Visual status**				
Normal sighted	9	22	10	26.0
Refractive errors	11	27	09	23.0
Visual impairment	12	29	11	28.0
Blindness	9	22	9	23.0

The psychometric properties of the Sepedi version of the CVF-QoL instrument were evaluated. The domains were averaged, and the Cronbach alpha test was conducted. The overall alpha score for Sepedi version 1.1 was 0.927, which indicates excellent internal consistency. In contrast, version 2.1 had an alpha score of 0.762, considered to demonstrate acceptable internal consistency, as shown in Table 4.

**TABLE 4:** Alpha coefficients and standard deviations for both Sepedi Versions of the Children’s Visual Function Quality of Life Instrument.

Scale or domain	Sepedi Version 1.1 of CVF-QoL				Sepedi Version 2.1 of CVF-QoL			
	Number of items averaged	Mean	SD	Alpha	Number of items averaged	Mean	SD	Alpha
School and learning	17	55 660	10 886	0.896	13	68 295	16 389	0.884
Mobility and orientation	8	66 150	10 746	0.902	7	65 038	12 029	0.891
Daily living skills	17	73 529	12 992	0.907	10	75 781	16 652	0.864
Hobbies, leisure and sport	9	56 566	12 050	0.889	8	65 441	16 691	0.844
Social interaction	6	81 250	7984	0.699	2	91 667	18 596	0.129
Psychological or emotional function	17	64 118	16 676	0.842	9	64 815	20 833	0.411
Treatment	8	70 980	16 135	0.638	8	81 250	9161	0.405
Sociocultural	7	79 762	9058	0.778	6	81 730	12 705	0.778
Overall	89	59 140	20 240	0.930	63	61 190	21 543	0.762

SD, standard deviation; CVF-QoL, Children’s Visual Function Quality of Life.

All learners submitted the questionnaire, which they completed at home 10 days after the initial attempt. Table 5 presents the results of the first and second attempts for Sepedi version 1.1 of the CVF-QoL, while Table 6 summarises the results for Sepedi version 2.1 of the same instrument. The overall Cronbach’s alpha scores for version 1.1 in the first and second attempts were 0.930 and 0.927, respectively, both indicating excellent internal consistency. Slightly lower overall Cronbach’s alpha scores for version 2.1 in the first and second attempts were obtained, that is, 0.762 and 0.712, respectively; however, both indicate moderate to good internal consistency (Maria et al. 2017).

**TABLE 5:** Cronbach’s alpha score for the first and second attempts of Sepedi version 1.1.

Sepedi version 1.1		First-attempt ANOVA results			Second-attempt ANOVA results		
Domain	Number of items	Mean	SD	Alpha	Mean	SD	Alpha
SL	16	55 660	10 886	0.896	55 560	10 857	0.884
MO	8	66 150	10 746	0.902	66 010	10 334	0.914
DLS	18	73 529	12 992	0.907	72 129	11 927	0.911
HLS	8	56 566	12 050	0.889	57 320	12 014	0.879
SI	7	81 250	7984	0.699	80 691	8105	0.706
PEF	17	64 118	16 676	0.842	63 970	16 514	0.843
T	8	70 980	16 135	0.638	70 980	16 135	0.638
SC	7	79 762	9058	0.778	79 762	9058	0.778
Overall	89	59 140	20 240	0.930	59 270	20 364	0.927

SL, school and learning; MO, mobility and orientation; DLS, daily living skills; HLS, hobbies, leisure and sport; SI, social interaction; PEF, psychological or emotional function; T, treatment; SC, sociocultural; SD, standard deviation; ANOVA, analysis of variance.

**TABLE 6:** Cronbach’s alpha score for the first and second attempts of Sepedi version 2.1.

Sepedi version 2.1		First-attempt ANOVA results			Second-attempt ANOVA results		
Domain	Number of items	Mean	SD	Alpha	Mean	SD	Alpha
SL	16	68 295	16 389	0.884	67 880	277 320	0.887
MO	8	65 038	12 029	0.891	64 850	151 613	0.891
DLS	18	75 781	16 652	0.864	75 156	273 680	0.853
HLS	8	65 441	16 691	0.844	65 441	278 670	0.844
SI	7	91 667	18 596	0.129	91 667	18 596	0.129
PEF	17	64 815	20 833	0.411	64 815	20 833	0.411
T	8	81 250	9161	0.405	81 250	9161	0.405
SC	7	81 730	12 705	0.778	81 730	12 705	0.778
Overall	89	61 190	21 543	0.762	60 595	21 502	0.712

SL, school and learning; MO, mobility and orientation; DLS, daily living skills; HLS, hobbies, leisure and sport; SI, social interaction; PEF, psychological or emotional function; T, treatment; SC, sociocultural; SD, standard deviation; ANOVA, analysis of variance.

In comparing the overall alpha scores for both versions across all attempts, Table 5 and Table 6 show that the alpha scores for version 1.1 are 99.7% correlated, and those for version 2.1 are 93.4% correlated. Therefore, the test–retest reliability for both versions demonstrates that they are reliable.

To ensure cross-cultural validation, the authors compared the Cronbach alpha scores of the Sepedi versions with those of the original English versions of the CVF-QoL. Detailed scores for the English version are available in another publication currently under review. As shown in Table 7, both the English and Sepedi version 1.1 demonstrated excellent internal consistency, as indicated by their Cronbach alpha scores. The same is true for both the English and Sepedi version 2.1, which also maintained a good level of internal consistency. Therefore, the translated version is valid and can be used with the same effectiveness as the English version.

**TABLE 7:** Comparison of Cronbach alpha scores between the original English versions and the translated Sepedi version of the Children’s Visual Function Quality of Life instrument.

Scale or domain	Ver 1.1 (α)		Ver 2.1 (α)	
	Eng	Sep	Eng	Sepedi
School and learning	0.862	0.896	0.881	0.884
Mobility and orientation	0.933	0.902	0.897	0.891
Daily living skills	0.927	0.907	0.888	0.864
Hobbies, leisure and sport	0.879	0.889	0.820	0.844
Social interaction	0.730	0.699	0.352	0.129
Psychological or emotional function	0.858	0.842	0.589	0.411
Treatment	0.734	0.638	0.681	0.405
Sociocultural	0.808	0.778	0.671	0.778
Overall	0.946	0.930	0.777	0.762

Eng Ver, English version of the CVF-QoL instrument; Sep Ver, Sepedi version of the CVF-QoL instrument; α, Cronbach alpha score.

## Discussion

Health-related quality of life (HRQoL) is a critical concept in healthcare, as it measures the impact of health conditions on patients’ well-being beyond traditional clinical indicators (Dahlmann-Noor et al. 2017; Khadka et al. 2010). The CVF-QoL instrument, which was translated, assesses various aspects of children’s lives, including school and learning, mobility and orientation, daily living skills, hobbies, leisure and sports, social interaction, psychological and emotional function, treatment and the sociocultural domain (Magakwe et al. 2025). Both the original English versions of the CVF-QoL demonstrated moderate to high internal consistency, indicating that the instrument reliably measures what it is intended to measure. While the CVF-QoL has been found to be valid and reliable in assessing the quality of life related to visual function for visually impaired children, it may not be easily applicable in the Sekhukhune setting, where the dominant language is Sepedi.

Translating HRQoL instruments is crucial for cross-cultural research and global clinical trials, particularly in diverse environments like South Africa (Abraham Lee, Kristina Monzon-Pajarillo & Pauline Santiago 2018; Geruschat et al. 2015). In this study, the CVF-QoL instrument was translated from English to Sepedi for rural school-aged children. This translation was essential to ensure the reliability of the CVF-QoL instrument in assessing the quality of life related to visual function among these children. Reliability refers to the ability of an instrument to consistently measure a construct, regardless of time, individuals or situations (Abraham Lee et al. 2018). Therefore, administering an instrument directly to a population with a different setting, culture and language to that in which the instrument was developed may not necessarily yield reliable results.

The study utilised the forward-backward-forward translation method, which is widely recommended (Abraham Lee et al. 2018; Elsman et al. 2019; Pérez-Mañá et al. 2019). Three professionals were involved in translating the instrument. Thirty targeted children confirmed that the content of both versions of the CVF-QoL was clear, comprehensive and reliable. This approach to face validation was also employed by Angeles-Han et al. (2011) while validating a vision-related quality-of-life instrument for children aged 8 to 18 years with juvenile idiopathic arthritis-associated uveitis (Angeles-Han et al. 2011). The study utilised a targeted population to assess whether the questionnaire effectively measured its intended construct. The CVF-QoL instrument demonstrated good internal consistency for both Sepedi version 1.1 and version 2.1, as measured by Cronbach’s alpha scores. This internal consistency for over 0.7 indicates that both the Sepedi versions of the CVF-QoL instrument measure what was intended to measure.

The CVF-QoL instrument involved children in its development, particularly during the initial phase of item generation. This involvement made it easier for children to understand the translated items of the CVF-QoL, as they originated from them. This understanding may have contributed to achieving this alpha value. The data collected after 10 days of completing the questionnaire show results comparable to the initial findings. These test–retest results indicate that the CVF-QoL is a stable instrument that is not influenced by the passage of time (Angeles-Han et al. 2011; Cochrane et al. 2011; Gothwal et al. 2003; Khadka et al. 2010).

The English versions of the CVF-QoL instrument are comparable to the Sepedi versions of the CVF-QoL instrument, further validating the translated versions. The social interaction component of version 2.1 contains only two items, which has resulted in a comparatively lower Cronbach alpha score. Research shows that having fewer items within a domain can lead to a lower alpha score (Maria et al. 2017). To address this issue, four items were adopted from the social interaction domain of the already validated version 1.1. This change increases the number of items in version 2.1 from 63 to 67, and it will now be referred to as version 2.2, attached as Online Appendix 3, supporting information S1. The adopted items are listed in Sepedi as follows:

- 64. Batho ba tenega ka wena ka lebaka la pono ya gago
- 65. Go lebelelana ka mahlong ge o boledišana le mogwera wa gago
- 66. Go tšea karolo ka ditiragalong tša go fapafapana (bjalo ka ditiragalo tša dipapadi) le bagwera
- 67. Go dira bagwera

There were some words that the translators struggled to translate into Sepedi. However, bringing the translators and PIs together in a Microsoft Teams meeting helped to reach an agreement. One example of a challenging translation was the phrase ‘Does not apply to me’, which could be translated as ‘Ga e šome go nna’ or ‘Ga ke amege’. The agreed-upon option was ‘Ga ke amege’. Another challenge involved words that were synonymous, such as ‘Raloka’ and ‘Bapala’. The choice between these words depended on the area or part of Limpopo province a person was coming. Ultimately, the authors relied on the children’s responses who reviewed the items during the piloting testing stage, and they chose the option ‘Raloka’.

The translation of the CVF-QoL instrument aligns with the recommendations for translating HRQoL tools into South African languages, as outlined by Marsh et al. (Marsh & Truter 2021). This process is similar to translating a VRQoL instrument from English to Filipino (Abraham Lee et al. 2018). Although the CVF-QoL is the first instrument to be translated into a South African language, several health-related tools have already been translated into one or more South African languages (Feeny et al. 2012; Kolisa & Van Wyk 2020; Wissing et al. 2010).

### Limitations and recommendations

Because of limited resources, the authors were unable to translate the CVF-QoL instrument into braille. As a result, blind children required assistance to complete the questionnaire. Each child was provided with a trained research assistant to help them with this process. The authors recommend that the CVF-QoL be translated into braille. It is further recommended that the CVF-QoL be translated into all of the official languages in South Africa to optimise its usage by all rural children in South Africa. It can also be modified and validated for an urban context and or causes of VI. Additionally, it should be validated across different communities and demographic groups.

## Conclusion

The Sepedi version of the CVF-QoL instrument developed for measuring the quality of life related to visual function in rural, school-going children demonstrated good internal consistency. The translation and initial validation of the CVF-QoL into Sepedi represents a meaningful step towards inclusive health measurement in South Africa. This instrument has the potential to elicit greater insight into the daily experiences of rural South African children living with VI, which can be used to guide the development of future health and education policies to benefit this vulnerable population.
